# The importance of additional intracranial injuries in epidural hematomas: detailed clinical analysis, long-term outcome, and literature review in surgically managed epidural hematomas

**DOI:** 10.3389/fsurg.2023.1188861

**Published:** 2023-08-01

**Authors:** Franz Marhold, Romana Prihoda, Philip Pruckner, Vanessa Eder, Anna Glechner, Irma Klerings, Jozsef Gombos, Branko Popadic, Anna Antoni, Camillo Sherif, Florian Scheichel

**Affiliations:** ^1^ Karl Landsteiner University of Health Sciences, Krems, Austria; ^2^ Division of Neurosurgery, University Hospital St. Poelten, St. Poelten, Austria; ^3^ Department for Evidence-Based Medicine and Evaluation, Danube University Krems, Krems, Austria; ^4^ Department of Urology, General Hospital Wiener Neustadt, Wiener Neustadt, Austria; ^5^ Department of Orthopedics and Trauma Surgery, Medical University of Vienna, Vienna, Austria

**Keywords:** traumatic brain injury, epidural hematoma (EDH), long-term outcome, extended Glasgow outcome score, additional intracranial injuries

## Abstract

**Objective:**

Epidural hematomas (EDH) occur in up to 8.2% of all traumatic brain injury patients, with more than half needing surgical treatment. In most patients suffering from this perilous disease, good recovery with an excellent clinical course is possible. However, the clinical course is mainly dependent on the presence of additional intracerebral injuries. Few studies comparing isolated and combined EDH in detail exist.

**Methods:**

We performed a retrospective single-center study from April 2002 to December 2014. The mean follow-up time was more than 6 years. In addition to analyzing diverse clinicoradiological data, we performed a systematic literature review dealing with a detailed comparison of patients with (combined) and without (isolated) additional intracerebral injuries.

**Results:**

We included 72 patients in the study. With increasing age, combined EDH had a higher incidence than isolated EDH. The mortality rate of the patients in the cohort was 10%, of which 0% had isolated EDH and 10% had combined EDH. Good recovery was achieved in 69% of patients, of which 91% had isolated EDH and 50% had combined EDH. A subgroup analysis of the different additional intracerebral injuries in combined EDH demonstrated no significant difference in outcome. A systematic literature review only identified six studies. Patients with isolated EDH had a statistically significantly lower mortality risk [relative risk (RR): 0.22; 95% CI: 0.12–0.39] and a statistically significantly lower risk of unfavorable Glasgow outcome scale score (RR: 0.21; 95% CI: 0.14–0.31) than patients with combined EDH.

**Conclusions:**

An excellent outcome in patients with surgically treated isolated EDH is possible. Furthermore, patients with combined EDH or isolated EDH with a low Glasgow coma scale (GCS) score may have favorable outcomes in 50% of the cases. Therefore, every possible effort for treatment should be made for this potentially lethal injury.

## Introduction

Epidural hematomas (EDH) occur in approximately 2.7%–8.2% of all traumatic brain injury (TBI) patients (1, 2). Even though this injury pattern represents a severe head injury, 12%–42% of patients are categorized as mild or moderate TBI (1). On the other hand, approximately 22%–56% present with coma on admission or immediately before surgery (1). This discrepancy may be explained by the severity of the trauma, the injury pattern, and the compartment in which EDH develops. Skull fractures with bleeding of the diploic veins, rupture of the middle meningeal artery or vein, or bleeding of venous sinuses mainly cause hematoma accumulation within the epidural space (3). Consequently, EDH is an injury of the skull than the brain, especially in isolated EDH without additional brain injuries.

In many cases, the clinical course of this presumably perilous disease may be benign if diagnosis and treatment are appropriate and rapidly set, particularly if no additional intracerebral injuries are present. However, these potential additional intracerebral injuries vary considerably in their extent and location, depending on the severity of the trauma. It is assumed that patients with additional intracranial injuries have a worse clinical course due to the additional injury to the brain. Earlier studies found additional intracerebral injuries present in 33% of injuries with subsequently significant adverse outcomes within this subgroup (4–6). It is of utmost importance to note the different prognoses of EDH with additional intracranial injuries for decision-making, therapeutic considerations, and the discussion of possible prognoses with relatives. A recent systematic review calculated that 3.1 million people require surgery for an EDH every year, showing clearly that this disease is a major global burden (2).

Therefore, we performed an in-depth analysis of patients with an EDH treated surgically. We specifically compared isolated EDH (EDH without additional intracerebral injuries) with so-called combined EDH (EDH with additional intracerebral injuries). Moreover, within the combined EDH group, we analyzed subgroups depending on the type of additional intracerebral injuries.

By presenting this consecutive case series, we aim to contribute important clinical information and outcome data for evidence-based decision-making and therapeutic considerations in the rather simple surgical procedure of hematoma evacuation in EDH. To the best of our knowledge, the literature still lacks sufficient information on detailed EDH analysis in the last 20 years.

## Methods

This study was performed at the Department of Neurosurgery of the University Hospital of St. Poelten (Karl Landsteiner University of Health Sciences), a level-one trauma center. We performed a retrospective cohort study including a prospective part with a survey conducted. The Local Ethics Committee of the Federal State of Lower Austria approved this study (GS4-EK-4/271-2014).

### Patient characteristics

We collected demographic, radiological, and clinical data, including age, gender, time of surgery, severity of TBI, Glasgow coma scale (GCS) score, pupillary status, polytrauma, surgical method, concomitant cranial lesions [fracture, traumatic subarachnoid hemorrhage (tSAH), intracerebral hematoma, and subdural hematoma (SDH)], localization of the EDH, etiology of the EDH, size of the EDH, monitoring of intracranial pressure (ICP), time period of ICP monitoring, duration of stay at intensive care unit (ICU) and hospital with/without complications, occurrence of sequelae, and—as outcome measure—assessment of extended Glasgow outcome scale (eGOS) score at discharge and follow-up (eGOS F/U). The mean follow-up time was 74 months (range 22–149 months).

The inclusion criteria included all patients, regardless of their age or sex, that were admitted to our hospital between April 2002 and December 2014 with a cranial EDH detected via cranial computed tomography (CCT) which required immediate neurosurgical hematoma evacuation by craniotomy or craniectomy. Patients with EDH treated non-surgically, burr hole hematoma evacuation, and spinal EDH were excluded.

Data were collected and evaluated using the hospital documentation system for each study participant. The neurologic outcome was assessed using the eGOS score at discharge and at follow-up (F/U) *via* a structured telephone interview, as published before (7, 8).

#### Definition of isolated and combined epidural hematoma

The patients were divided into two main groups, namely, isolated EDH and combined EDH. An isolated EDH was defined as an EDH without significant additional intracerebral injuries. This group could include patients with skull fractures without depression or skull base fractures. Combined EDH was defined as EDH including additional intracerebral lesions such as tSAH, SDH, intracerebral hemorrhage (ICH), cerebral contusions (CONT), and depressed calvarial fractures (DF).

To further analyze subgroups within the combined EDH group, we defined three groups: group 1 consists of EDH with either tSAH or ICH but without additional SDH, group 2 consists of EDH with either tSAH or ICH and with additional SDH, and group 3 consists of EDH, tSAH, ICH, and SDH combined.

### Systematic literature review

#### Literature search

**Figure 1:**
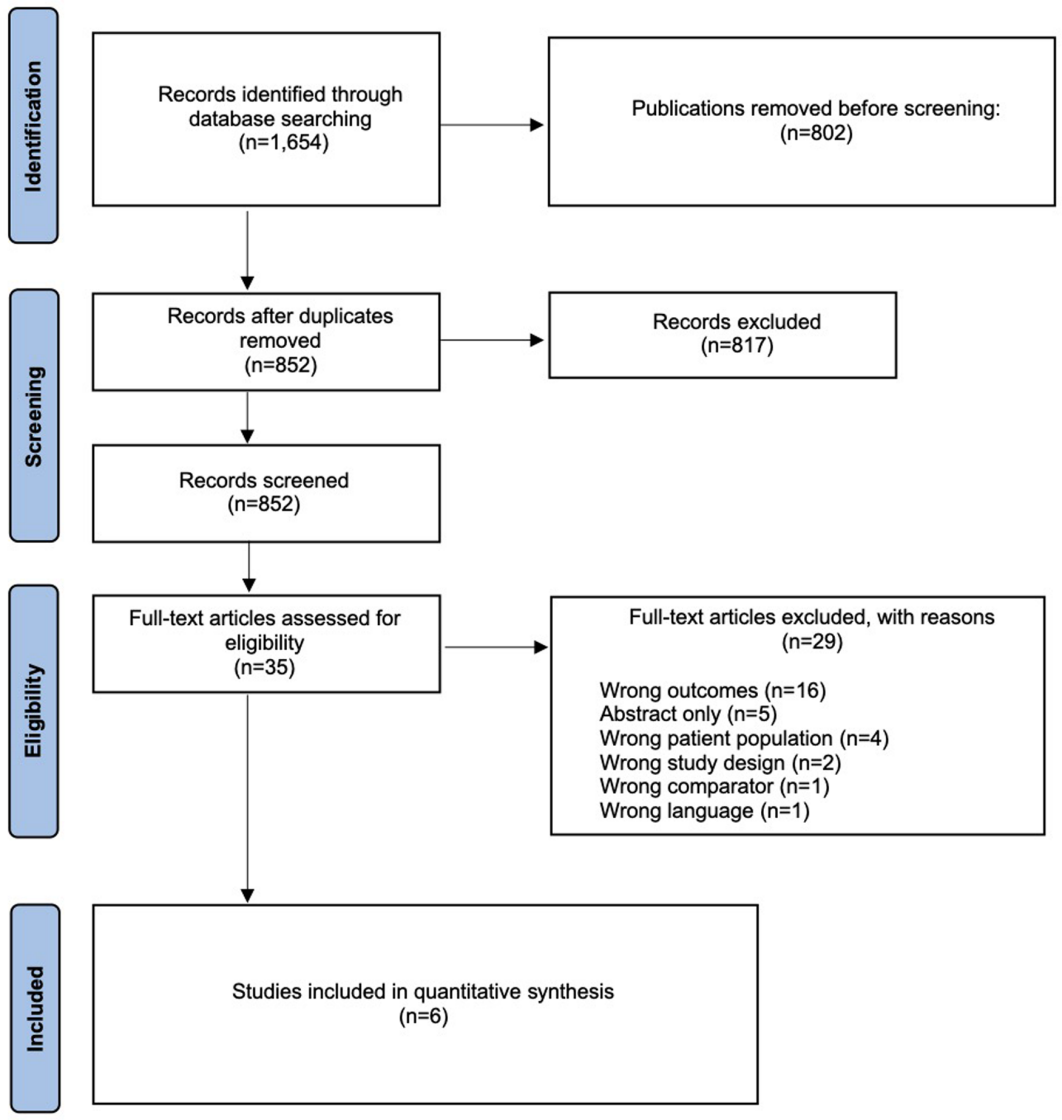
PRISMA flow diagram for selection of studies based on inclusion during systematic review.

We performed a systematic literature search on published articles through 15 February 2021 in the databases Ovid MEDLINE, Cochrane Library, ClinicalTrials.gov, DynaMed, and UpToDate. Figure 1 presents the PRISMA flow diagram. The Supplementary file *literature research* presents the complete search strategy.

#### Study selection

Two researchers independently reviewed abstracts and full-text articles. The eligibility criteria for studies were defined *a priori* and are presented in Supplementary material. We included systematic reviews and meta-analyses, randomized controlled trials, and prospective or retrospective cohort studies that analyzed the neurological outcome of patients with EDH, with or without additional intracranial injury.

#### Data extraction and quality assessment

Trained reviewers extracted data from each study and assessed the risk of bias. A senior reviewer checked the data for accuracy and independently assessed the risk of bias classification. We used the ROBINS-I tool to assess the risk of bias in prospective and retrospective cohort studies. The overall risk of bias in the studies was classified as low, unclear, or high.

#### Data synthesis and analyses

We performed meta-analyses when three or more studies provided data for quantitative analysis and were similar with respect to populations studied. We calculated the relative risk (RR) for an unfavorable Glasgow outcome scale (GOS) score and mortality after surgery. We performed a heterogeneity test (*I*^2^ statistic, Cochran's *q* test) for each meta-analysis and applied DerSimonian and Laird's method for random-effects models. We performed sensitivity analyses, excluding studies at high risk for bias. When there was a large heterogeneity (*I*^2^ statistic >60%), we examined the reasons for heterogeneity using meta-regressions. Because of the small number of studies identified, we did not assess publication bias. All statistical analyses were performed using Review Manager 5.4.

#### Grading the quality of evidence

One reviewer assessed the quality of evidence for each outcome of interest using an approach proposed by the Grading of Recommendations Assessment, Development, and Evaluation (GRADE) Working Group (9). A second senior reviewer checked the assessment. The assessment of the certainty of evidence for each outcome included the risk of bias, inconsistency, indirectness, imprecision, and reporting bias. The strength of evidence was categorized into four levels, namely, high, moderate, low, and insufficient. We discussed disagreements regarding the grading and resolved them by consensus.

### Statistical analysis

The software SPSS Statistics, version 27.0 (IBM Corp., Armonk, NY) was utilized for statistical analyses. Testing for normal distribution was performed using the Kolmogorov–Smirnov test. Normally distributed metric data were reported using the mean and standard deviation, whereas skewed data were summarized using the median and range. Categorical data were presented as absolute frequencies and percentages to characterize the patient cohort. A *T*-test was performed on normally distributed variables, whereas the χ^2^ test was used for dichotomous variables. A *p*-value of <0.05 was considered statistically significant.

## Results

In total, 72 patients were included in this study and sorted into two groups, namely, isolated EDH (*n* = 32) and combined EDH (*n* = 40). The systematic literature search only identified six papers (Figure 1), differentiating the outcome of patients with isolated (patients with EDH with/without non-displaced skull fractures, skull base fractures) and combined (combination with other intracranial injuries: subarachnoid hemorrhage, acute SDH, contusions, traumatic ICH, and displaced fractures) EDH, of which five could be used for the pooled analysis (5, 6, 10–13).

### Clinical characteristics

**Figure 2:**
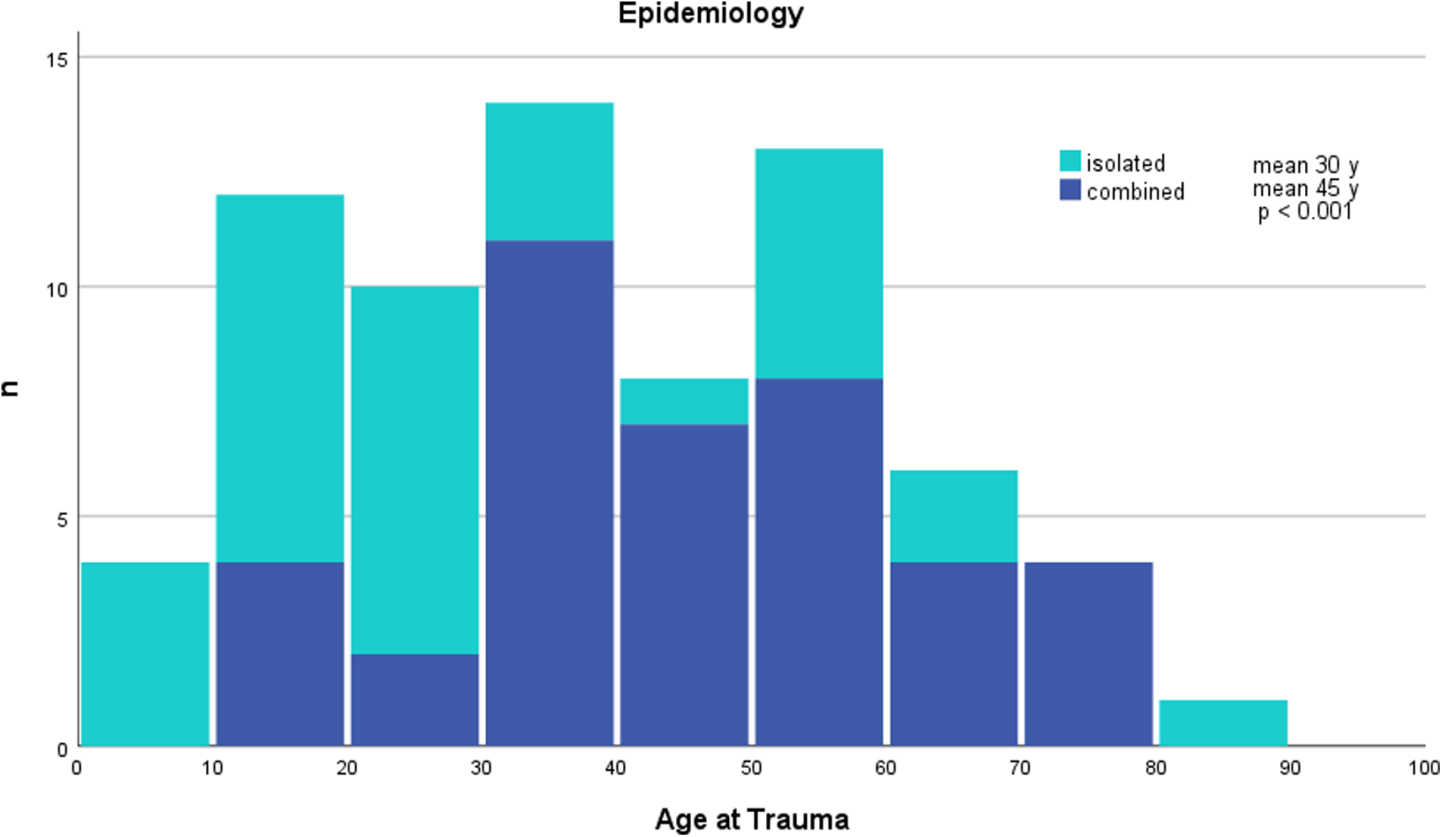
Age distribution at the time of the EDH.

Gender was distributed to 22 males (69%) and 10 females (31%) in the isolated EDH group and 34 males (85%) and 6 females (15%) in the combined EDH group. The mean age of patients was significantly higher in the combined EDH group (45.4 years vs. 30.3 years, *p* = 0.001), as demonstrated in Table 1. In general, either isolated or combined EDH was present in all age groups, with a peak for isolated EDH in the second and third decades and a peak for combined EDH in the fourth, fifth, and sixth decades (Figure 2). The incidence in the geriatric patient group (age above 65 years) decreased continuously.

**Table 1:** Clinicoradiological characteristics.

	No. of patients *n* (%)			*p*-value (*α *= 0.05)	
	Total	Isolated	Combined		
*n*	72 (100)	32 (44)	40 (56)		
Sex ratio (female:male)	1:3,5	1:2,2	1:5,7	ns	
Male	56 (78%)	22 (36%)	34 (47%)		
Female	16 (22%)	10 (14%)	6 (8%)		
Mean age (years)	39	30	45	*p *= 0.001	*T*-Test od. ANOVA
Range	4–80	4–80	10–78		
Clinics					
Mild TBI	34 (47%)	20 (59%)	14 (41%)	*p *= 0.02	Chi-Quadrat
Moderate TBI	12 (17%)	1 (8%)	11 (92%)	*p *= 0.006	Chi-Quadrat
Severe TBI	26 (36%)	11 (42%)	15 (58%)	ns	
Anisocory	13 (100%)	5 (38%)	8 (62%)	ns	1 missing
Lucid interval	11 (100%)	7 (64%)	4 (36%)	ns	2 missing
Polytrauma	22 (100%)	10 (45%)	12 (55%)	ns	
Imaging					
Supratentorial region of hematoma in relation to calvarial bone	72 (100%)	32 (44%)	40 (56%)	ns	
One region	41 (57%)	17 (41%)	24 (59%)		
With infratentorial involvement	3 (4%)	2 (67%)	1 (33%)		
Two regions	18 (25%)	7 (39%)	11 (61%)		
Three regions	10 (14%)	6 (60%)	4 (40%)		
Hematoma diameter mean (mm)	19.6	20,8	18,6	ns	
Range (mm)	2–50	2–50	5–50		
Midlineshift (n)	42 (100%)	24 (57%)	18 (43%)	*p *= 0.01	Chi-Quadrat
Mean (mm)	7.5	6,7	8,6	ns	
Range (mm)	2–20	2–15	2–20		
Patients with documented source of hemorrhage	69 (100%)	31 (45%)	38 (55%)		
Detected bleeding sources	73 (100%)	31 (42%)	42 (58%)		
Medial meningeal artery	26 (38%)	13 (42%)	13 (31%)	ns	
Fracture hematoma	33 (48%)	16 (52%)	17 (40%)	ns	
Sinus hemorrhage	14 (20%)	2 (6%)	12 (29%)	*p *= 0.01	Chi-Quadrat
Multiple sources	4 (6%)	0	4 (10%)	ns	

Combined EDH: 42 detected sources of hemorrhage in 38 patients, 2 missing values, 4 patients had two sources of hemorrhage retrospectively; isolated EDH: 31 detected sources of hemorrhage, 1 missing value.

According to the GCS, TBI severity was classified as mild in 34 patients, moderate in 12 patients, and severe in 26 patients. A statistically significant difference in the distribution of the TBI classification between groups was seen, with more patients with isolated EDH having a mild TBI (*p* = 0.02) and with more patients with combined EDH having a moderate TBI (*p* = 0.006). No significant difference for isolated or combined EDH could be detected within the severe TBI group. There was also no significant difference within the groups regarding anisocoria and polytrauma. The quantity of categorized polytrauma was almost equally distributed (isolated 45%, 10/22; combined 55%, 12/22, Table 1).

### Radiological characteristics

There was no statistically significant difference observed in the distribution of EDH locations. The mean maximal hematoma diameter showed no significant difference. Midlineshifting appeared significantly more often in patients with isolated EDH than in those with combined EDH (*p* = 0.01), although the mean diameter of the midline shift did not reach a statistical difference.

Sinus hemorrhage as the source of bleeding was found more often in the combined EDH group than in the isolated EDH group (*n* = 12, 86% vs. *n* = 2, 14%, *p* = 0.01, Table 1). No other significant differences with regard to the source of bleeding were observed between the two groups.

Concerning isolated EDH, the source of hemorrhage was detected in 31 patients, with fracture hematoma being the most frequent source (52%, 16/31), followed by middle meningeal artery rupture (42%, 13/31) and sinus hemorrhage (6%, 2/31).

Concerning combined EDH, the source of hemorrhage was detected in 42 patients, with fracture hematoma in 40% (17/42), middle meningeal artery rupture in 31% (13/42), and sinus hemorrhage in 29% (12/42). A total of four patients presented with multiple sources of hemorrhage (Table 1).

The characteristics of additional intracerebral lesions included SDH, SAH, ICH or CONT, CF, DF, BSF, or any combination of them. Within the combined EDH group, various combinations of the intracerebral lesions were found, which we grouped into three subgroups (groups 1, 2, and 3). These subgroups were distributed almost equally, with 38% in group 1, 31% in group 2, and 31% in group 3 (Table 2).

**Table 2:** Subgroup analysis of outcome in combined EDH.

Outcome subgroup analysis	No. of patients *n* (%)			*p*-value
	Group 1	Group 2	Group 3	
*n *= 39/40	15 (38)	12 (31)	12 (31)	
eGOS discharge (mean)	4.5	3.5	3.8	ns
eGOS F/U (mean)	5.9	5.1	5	ns

Grouping includes only subgroups of combined EDH. Group 1 consists of additional tSAH and/or ICH, group 2 consists of additional SDH with tSAH or ICH and group 3 consits of additional tSAH, ICH and SDH 2 missing at F/U.

### Therapeutic modalities

Concerning the timing of surgery, no statistically significant difference was found. In general, the majority of patients in both groups were treated with craniotomy (76%); in patients with combined EDH, craniectomy was performed significantly more often (82% vs. 18%, *p* = 0.01). There were significant differences in the need for an ICP probe (28% vs. 72%, *p* = 0.002), the number of complications at the ICU (20% vs. 65%, *p* < 0.001), the time of extubating (1.5 days vs. 8.0 days, *p* = 0.021), and the time spent at the ICU (4.6 days vs. 16.2 days, *p* < 0.001). The ICP measuring time (7.7 days vs. 13.8 days, *p* = 0.056) showed a trend toward longer measuring time in combined EDH. Table 3 provides additional details.

**Table 3:** Therapeutic modalities.

Therapeutic modalities	No. of patients *n* (%)			*p*-value
	Total	Isolated	Combined	
Time of day	67 (100%)	30 (45%)	37 (55%)	ns
Day (7–22 h)	44 (66%)	21 (31%)	23 (34%)	
Night (22–7 h)	23 (34%)	9 (13%)	14 (21%)	
Procedure	72 (100%)	32 (44%)	40 (56%)	
Craniotomy	55 (76%)	29 (91%)	26 (65%)	
Craniectomy	17 (24%)	3 (9%)	14 (35%)	*p *= 0.01
Intensive care management				
ICP probe	36 (50%)	10 (28%)	26 (72%)	*p *= 0.002
Number of complications at ICU	28 (39%)	6 (20%)^a^	22 (65%)^b^	<0.001
Frequency of hospital visits due to sequelae	31 (43%)	14 (48%)^c^	17 (60%)^d^	ns
Metric ICU parameters				
ICP measuring time (mean in days)	12.1	7.7	13.8	*p *= 0.056
Standard deviation	8.6	4.9	9.2	
Range	1–36	1–15	2–36	
Time of extubation (mean in days)	4.5	1.5	8.0	*p *= 0.021*
Standard deviation	9.7	4.0	13.2	
Range	0–55	0–18	0–55	
Tracheotomies excluded (*n*)	14	3	11	
Time spent at ICU (mean in days)	10.8	4.6	16.2	*p *<* *0.001**
Standard deviation	13	6.7	14.7	
Range	0–50	0–23	0–50	
Time spent at primary hospital (mean in days)	24.2	14.3	33.3	*p *<* *0.001***
Standard deviation	23	10.1	27.5	
Range	2–102	2–49	4–102	
Discharge to other hospital	31	10	21	*p *= 0.018****

^a^ 6/30.

^b^ 22/34.

^c^ 14/29.

^d^ 17/28.

* *n *= 48, 15 patients excluded (14 tracheostomy, 1 dead), 9 patients missing.

** *n *= 65, 1 patient excluded (dead), 6 patients missing.

*** *n *= 67, 1 patient excluded (dead), 4 patients missing.

**** *n *= 67, 1 patient excluded (dead), 4 patients missing.

### Outcome

**Figure 3:**
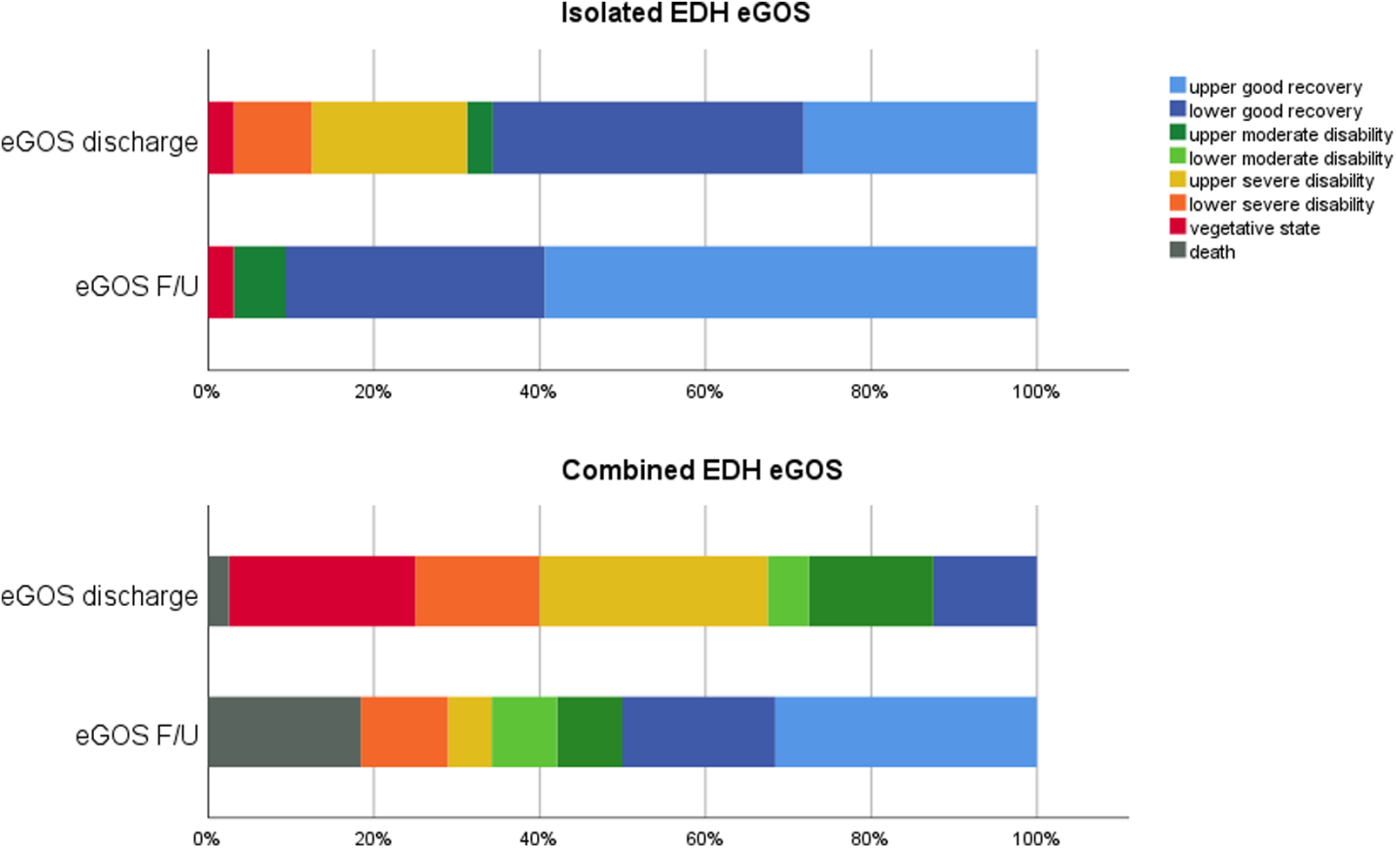
Outcome in isolated and combined EDH at discharge and F/U.

**Figure 4:**
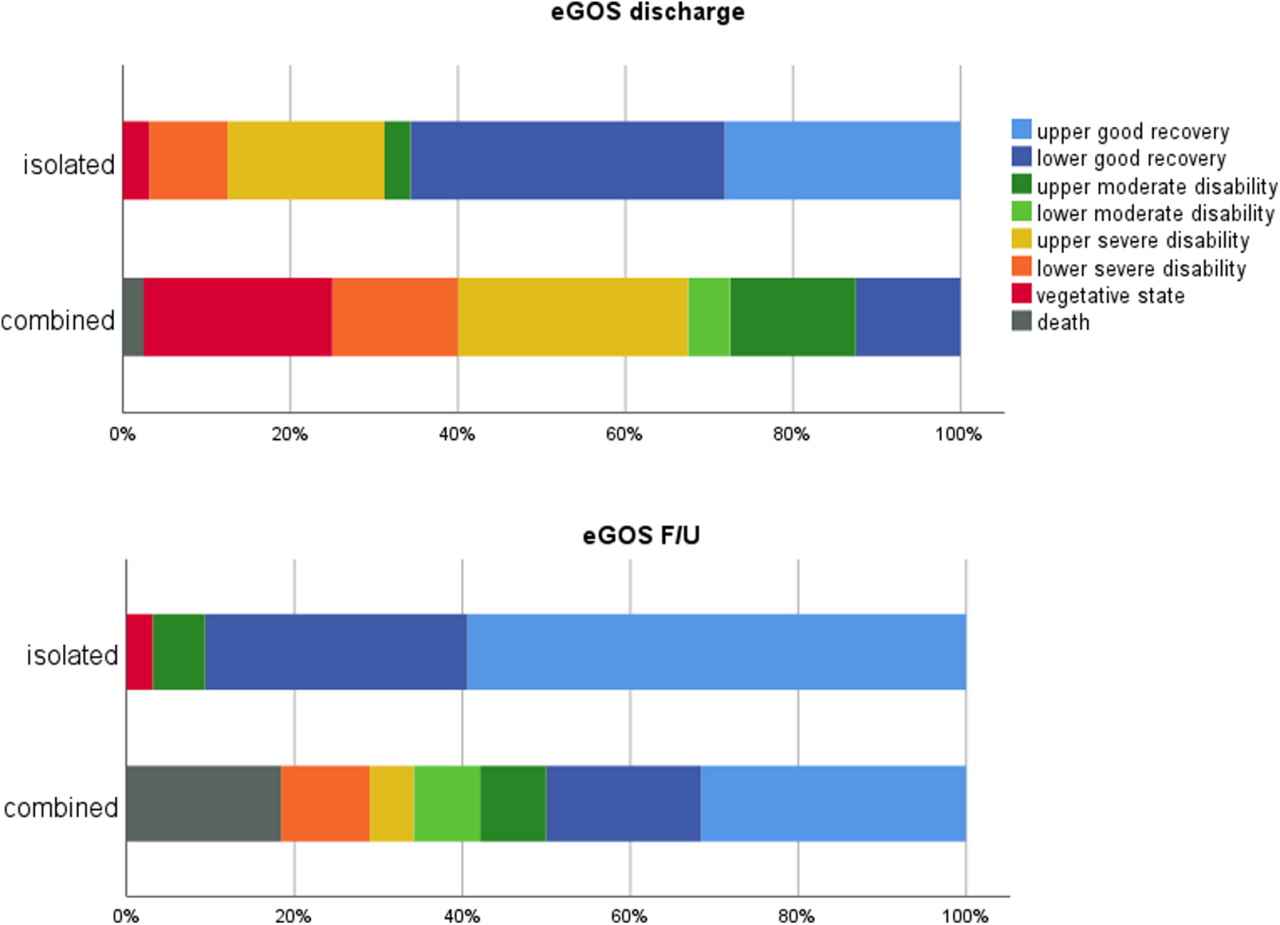
Outcome at discharge and F/U in isolated and combined EDH.

The clinical outcome was assessed using the eGOS at two time points, namely, discharge and F/U (Figures 3, 4). The mean F/U time was 6.2 years. According to the eGOS, we subdivided our outcome analysis into mortality, disability, and good recovery (Table 4).

**Table 4:** Outcome data using the eGOS.

Outcome	No. of patients *n* (%)						*p*-value
	Total		Isolated		Combined		
	Discharge	F/U	Discharge	F/U	Discharge	F/U	
*n*	72 (100)	70 (100)	32 (44)	32 (46)	40 (56)	38 (54)	*p *< 0.0001*
Mortality (eGOS 1)	1 (1)	7 (10)	0	0	1 (3)	7 (18)	
Disability (eGOS 2-6)	45 (63)	15 (21)	11 (34)	3 (9)	34 (85)	12 (32)	
Good recovery (eGOS 7, 8)	26 (36)	48 (69)	21 (66)	29 (91)	5 (13)	19 (50)	
eGOS mean (1–8)	4.9	6.3	6.2	7.4	4.1	5.1	*p *< 0.0001**; *p *= 0.008***
Range			2–8	2–8	1–7	1–8	

2 missing F/U in combined EDH patients.

* eGOS discharge vs. F/U.

** eGOS discharge isolated vs. combined.

*** eGOS F/U isolated vs. combined.

The mean eGOS scores of the whole cohort were higher at F/U than at discharge (6.3 vs. 4.9, *p* < 0.0001). Comparing the eGOS scores of isolated and combined EDH patients, differences at discharge (*p* < 0.0001) and F/U (*p* = 0.008) reached statistical significance at both time points.

In total, one patient (1%) died before discharge, and seven patients passed away during the F/U period (10%). Mortality was exclusively observed in the combined EDH group. Survival in a vegetative state was observed in 10 patients at discharge, of whom one was in the isolated EDH group and nine patients were in the combined EDH group. At F/U, only one patient remained in a vegetative state.

Disability (eGOS 2–6) was observed in 63% (45/72) of the patients at discharge, which then decreased to 21% (15/70) at F/U. Within the isolated EDH group, disability decreased from 34% (11/32) to 9% (3/32), and within the combined EDH group, disability decreased from 85% (34/40) to 32% (12/38).

Good recovery (eGOS 7, 8) was seen in 36% (26/72) of the patients at discharge and increased to 69% (48/70) at F/U. Within the isolated EDH group, good recovery increased from 66% (21/32) to 91% (29/32), and within the combined EDH group, good recovery increased from 13% (5/40) to 50% (19/38).

In addition, we conducted a subgroup analysis to investigate differences in outcome within the subgroups of combined EDH. The mean eGOS score at discharge in groups 1, 2, and 3 was 4.5, 3.5, and 3.8, respectively. This increased to 5.9, 5.1, and 5.0, respectively, at F/U. There was no significant difference in outcome between the different subgroups of combined EDH.

### Literature search

**Figure 5:**
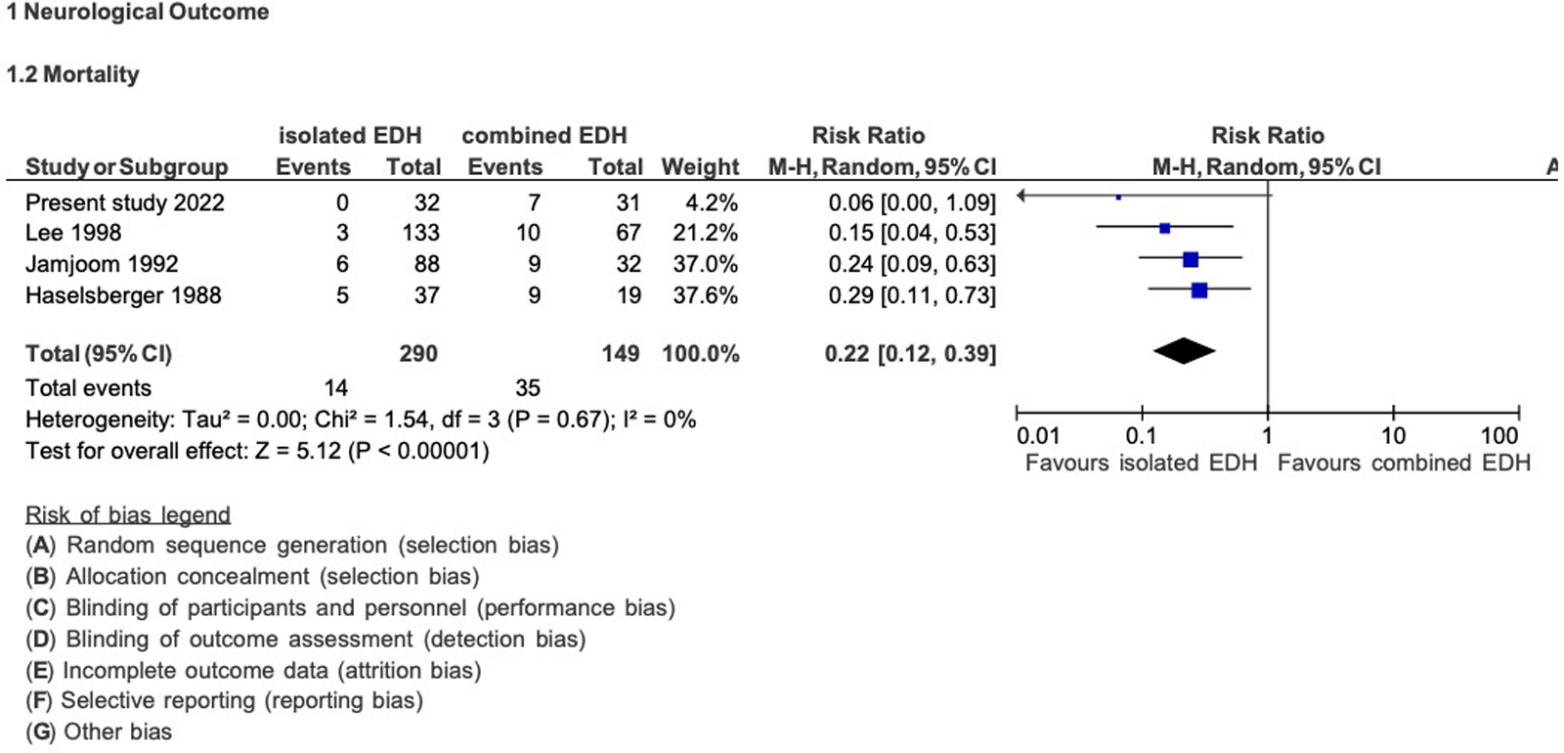
Pooled analysis of mortality data in EDH.

The systematic literature review included six studies for further analysis. For data on mortality, the results of four studies (including the present study) that met the inclusion criteria were pooled (Figure 5). Patients with isolated EDH had a statistically significantly lower mortality risk than patients with combined EDH (RR: 0.22; 95% CI: 0.12–0.39) (5, 10, 12). Among the patients with isolated EDH, 5% (14/290) died, while 24% (35/149) of patients with combined EDH experienced mortality.

**Figure 6:**
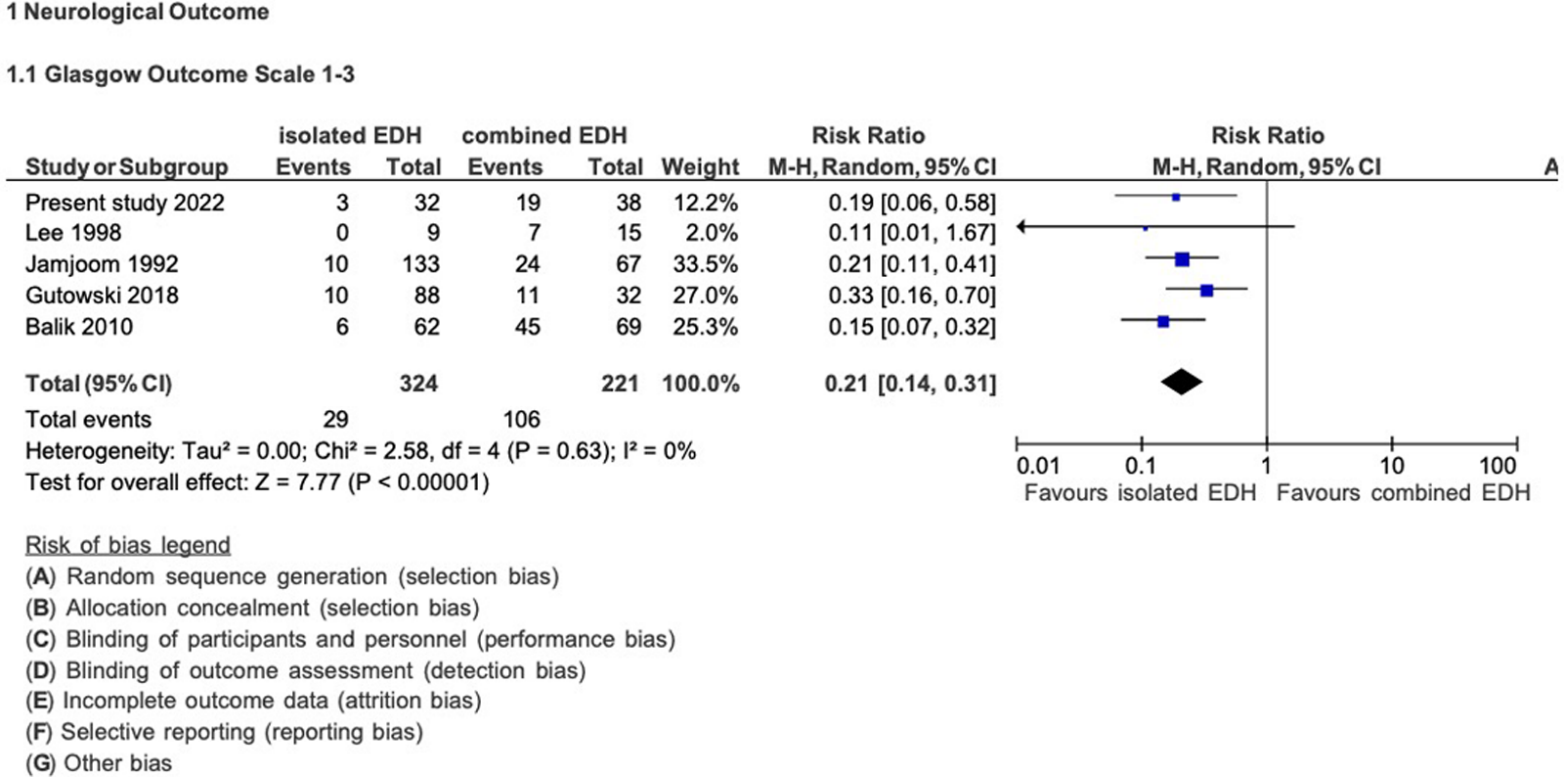
Pooled analysis of morbidity data (clinical outcome) in EDH.

In addition, we analyzed the clinical outcome data of five studies (including the present study, Figure 6) (5, 6, 10, 12, 14). Patients with isolated EDH had a statistically significantly lower risk of experiencing an unfavorable GOS score than patients with combined EDH (RR: 0.21; 95% CI: 0.14–0.31). Among patients with isolated EDH, 9% (29/324) developed an unfavorable GOS score, while 48% (106/221) of patients with combined EDH had an unfavorable GOS score.

## Discussion

In this study, we analyzed the differences in clinical, radiological, and outcome data of 72 patients with isolated and combined EDH (including additional intracerebral injuries) and within the subgroup of combined EDH.

In general, the therapeutic approach in EDH is straightforward, with hematoma evacuation and favorable prognosis in many patients. In particular, patients with isolated EDH without additional intracerebral injuries show favorable outcomes with low mortality (6). However, few studies clearly distinguish between isolated and combined EDH and their respective outcomes (6, 15). A systematic literature review identified only six papers (5, 6, 10–13, 16), of which three were published between 1988 and 1998 and the others within the last decade. They presented data from 755 patients with ages from 1 to 91 years.

Early identification of patients with assumed worse outcomes is of major clinical interest for further decision-making. Therefore, the aim of this study was to emphasize the differences between isolated EDH and combined EDH after a long-term follow-up assessment.

### Demographics

Historically, TBI predominantly affected male patients in the second or third decade of life. Demographics have significantly changed, and treatment improved over the last three decades (6, 17, 18). Thus, recent studies suggest a significant epidemiologic change with the establishment of a second age peak in TBI in geriatric patients (17, 19–22). In our cohort, we could not identify a second incidence peak for the whole cohort. Nevertheless, in isolated EDH, there are incidence peaks within the second and third decades, which subsequently decrease. The incidence of combined EDH significantly increased from the fourth to the sixth decades. This may be explained by different injury patterns associated with older age and the use of medication that affects hemostasis, which increases mortality and worsens outcomes, in general, in TBI patients (17, 23–25). Gutowski et al. reported that patients with combined EDH (vs. isolated EDH) were more frequently on anticoagulative (3% vs. 0%) and antiplatelet (16% vs. 3%) therapy than patients with isolated EDH (6). Nevertheless, patients with isolated EDH were significantly younger than patients with combined EDH (45.4 years vs. 30.3 years, *p* < 0.001). Recently, a cutoff point for good outcomes in EDH was identified for patients younger than 55 years (26). This could reflect not only better outcomes in younger age groups but also higher incidence of isolated EDH with a predictable better outcome. The increasing incidence of combined EDH with advancing age can be attributed to the fact that older age is considered a predictive factor for worse outcomes.

### Clinicoradiological features

We found significant differences in the distribution of mild and moderate TBI, but not severe TBI, within the groups. The GCS defines TBI severity, and its assessment should rather be performed continuously—as described originally—to early detect neurologic deterioration (27–29). We used the initial GCS at the scene and found significantly more patients with mild TBI in the isolated EDH group and significantly more patients with moderate TBI in the combined EDH group. This is in contrast to the recently published paper by Gutowski et al. showing no significant differences in TBI severity with almost equal distribution within the two groups (6). They showed a relatively higher proportion of mild TBI (65%), which is also higher than that observed in our cohort (47%). On the other hand, they recognized severe TBI in only 20% of the patients, compared with 36% in our cohort. The difference was generated due to the different time points in assessing the GCS, as Gutowski et al. assessed on admission and we assessed at the scene of the incident. In our series, no significant differences of isolated vs. combined EDH patients in severe TBI were recognized, as one would expect more patients with severe TBI within the combined EDH group. The absence of significant differences in anisocoria, a measure of brain injury severity, underlines this. The shortcomings of the GCS in multiple injured patients, with poor sensitivity in this subgroup, explains the absence of significant differences within the groups in polytraumatized patients (30). In general, the distribution of patients with a GCS <8 is between 17% and 52% (5, 6, 10, 13, 14), with one study even describing comatose patients in 63% (12).

Radiologically, only the incidence of the midline shift showed statistical differences between the groups. Interestingly, patients with isolated EDH developed shifting of the midline significantly more often, although the mean diameter of the shift was smaller than that in combined EDH. Additional intracerebral injuries may alter the pressure in the intradural compartment (subdural, parenchymatous) due to the evolution of intraparenchymal injuries with subsequent secondary brain damage. Theoretically, this increased intracerebral pressure may counteract the increased pressure in the epidural compartment that evolved due to the hematoma. Gutowski et al. also demonstrated this observation with an incidence of 69% midline shift (>5 mm) in isolated EDH and 54% midline shift in combined EDH, although differences did not reach statistical significance (6). However, a larger patient series is needed to prove this theory.

In 69 patients, 73 documented sources of bleeding were found, with four patients suffering from multiple sources. In the literature, the source of bleeding in EDH is almost always traced back to a (traumatic) rupture of the middle meningeal artery, and other sources are rarely reported (14). The presence of a hematoma with arterial bleeding is a highly unfavorable factor (31). A swirl sign, found as a sign of arterial bleeding on CCT scans, was correlated with a favorable outcome in 62%, as opposed to 85% of patients without it. Pruthi et al. found a mortality rate of more than 20% in mixed-density hematomas (32, 33). It is noteworthy and contradictory to the existing literature that in nearly half of the cases (48%, 33/69), the main source of bleeding was a fracture hematoma, while rupture of the middle meningeal artery was found in only 38% (26/69). Another interesting and important finding is that we found sinus hemorrhage significantly more often within the combined EDH group (86%, 12/14, *p* = 0.01), which might be associated with a stronger impact of the trauma to the skull.

### Therapeutic modalities

Significant differences in most therapeutic parameters reflect the increasing intensity of the management of patients with combined EDH.

### Outcome

Few studies report outcome data in surgically treated EDH in detail, which clearly contrasts outcome analyses in SDH (31, 34). Most studies only differentiate between favorable and unfavorable outcomes or use the GOS score. More specific data are reported, instead, in studies analyzing subgroups of patients with EDH, especially those with EDH in the posterior fossa (31). To the best of our knowledge, we did not find a study using the eGOS at two different time points. Taking into consideration that all grades of trauma, clinical outcomes, and cerebral injury severity information were included, the mortality rate of 10% at F/U of our cohort seems low. In their univariate analysis, Mejaddam et al. also demonstrated EDH as one predictor (besides others) of a high GOS score (35). This finding is consistent with the studies conducted by Leitgeb et al. and Choi et al., which reported mortality rates of 22.2% and 13.9%, respectively, specifically in patients with severe TBI with EDH (15, 34). Interestingly, in our series, mortality was only seen in patients with combined EDH and increased between discharge and F/U. The in-hospital mortality rate was only 1%, which is very low. As we included only patients with surgically treated EDH, the periprocedural mortality, therefore, seems neglectable for these patients. Nevertheless, in the long term, these injuries show a significant increase in the mortality rate.

Patients ending up with disability at F/U, defined in our cohort with an eGOS score of 2–6, also showed—as expected—a difference between isolated and combined EDH with 9% vs. 32%. However, the significant decrease in the number of disabled patients, from 85% at discharge to 32% at F/U, in combined EDH patients is noteworthy (Table 4). This finding supports attempting any efforts to perform post-discharge neurorehabilitation with substantial supposable improvement in long-term F/U.

Gutowski et al. demonstrated a favorable outcome of 30% in patients with concomitant intracranial injuries and 90% in patients with isolated EDH (6). In our series, we found a good recovery rate (eGOS 7, 8) at F/U in 50% of patients (19/38) with combined EDH and 91% of patients (29/32) with isolated EDH. Considering this, it is noteworthy that our definition of good recovery was the eGOS score of ≥7, which is strict and reflects patients who were able to return to their life before the injury. In almost all outcome analyses for any type of TBI, the GOS is used, and a favorable outcome is defined with a GOS score of 4 or 5. However, a GOS score of 4 means moderate disability with reduced work capacity and is somehow similar to the eGOS score of 6, which is defined as upper moderate disability and also reduced work capacity.

Within the combined EDH group, we performed a subgroup analysis for the detection of differences in outcome dependent on the variable concomitant intracerebral injuries. Yet, no statistically significant difference in the mean eGOS at discharge or F/U in these subgroups was found. As expected, in all three groups, the mean eGOS score increased within the time period from discharge to F/U. Therefore, we assume that the outcome in combined EDH is significantly affected by the presence of additional intracerebral injuries, but there seems to be no correlation between the distinct kind of these additional injuries and the outcome.

### Limitations

This study has some limitations. (1) The retrospective design must be considered a limitation. (2) The small patient number (72), with its underlying uncertainty of the statistical statements, is a major limitation, although very few studies reporting on the outcome in EDH in more than 100 patients exist. (3) The level of evidence of the studies that met the inclusion criteria of our systematic literature research was classified as moderate and low, and two of the studies were classified as having a high risk of bias because important factors influencing the outcome of surgery were not considered in the statistical analysis (5, 12). (4) One has to remember that only patients eligible for surgery were included, and we did not observe bilateral fixed pupils—a generally accepted predictor for adverse outcomes—in our cohort. However, considering the good clinical course in most patients with EDH, our in-house philosophy permits patients with bilateral mydriasis to be referred to surgery in selected cases.

## Conclusions

An excellent clinical outcome in patients with isolated EDH, with more than 90% experiencing good recovery (eGOS 7, 8), is possible. However, the outcome in traumatic EDH is significantly worse in patients with additional intracerebral injuries in the short and long terms. A good recovery rate at F/U of 50% observed in patients with combined EDH, who initially presented with severe TBI, still appears more favorable than that in other surgically treated patients with severe TBI. The type of additional intracerebral injuries, as defined in our subgroups, seems to have no significant impact on outcomes.

We, therefore, suggest that efforts should be made to facilitate optimal treatment prospects for these patients with EDH.

## Data Availability

The original contributions presented in the study are included in the article/**Supplementary Material**, further inquiries can be directed to the corresponding author.
