# Supplementary material for: The importance of additional intracranial injuries in epidural hematomas: detailed clinical analysis, long-term outcome, and literature review in surgically managed epidural hematomas
Source: Front Surg. 2023 Aug 1;10:1188861. doi: 10.3389/fsurg.2023.1188861 (PMC10427765; doi:10.3389/fsurg.2023.1188861)
Supplement: Supplementary file 1 [file Datasheet1.docx]

| Population | Adults (>18 years) with isolated EDH or in combination with other intracranial injuries   1. *“Isolated”: patients with EDH with/without non-displaced skull fractures / base fractures* 2. *“Combination with other intracranial injuries”: traumatic subarachnoid hemorrhage, acute subdural hematoma, contusions, traumatic intracerebral hemorrhage, displaced fracture* |
| --- | --- |
| Intervention | Surgery: craniotomy and hematoma evacuation; Burr hole evacuation, trepanation |
| Outcomes | Glasgow Outcome Scale (GOS), Glasgow Outcome Scale extended (GOSe), mortality, outcome after surgery |
| Study design | systematic reviews and meta-analyses  RCTs  prospective and retrospective cohort studies  health economic studies |

***Table X:*** *Eligibility criteria of included studies*
