# Supplementary material for: The importance of additional intracranial injuries in epidural hematomas: detailed clinical analysis, long-term outcome, and literature review in surgically managed epidural hematomas
Source: Front Surg. 2023 Aug 1;10:1188861. doi: 10.3389/fsurg.2023.1188861 (PMC10427765; doi:10.3389/fsurg.2023.1188861)
Supplement: Supplementary file 2 [file Datasheet2.docx]

Literature research

The search terms used were derived from the Medical Subject Headings System (MeSH) of the National Library of Medicine. In addition, free-text terms were used and a Pubmed-similar-articles search was performed. Publications whose abstracts had been identified as potentially relevant in the preliminary search served as source references. We limited electronic searches to “adult 19 years or older” and “human.”

Ovid MEDLINE(R) ALL 1946 to February 15, 2021

| **#** | **Searches** | **Results** |
| --- | --- | --- |
| 1 | Hematoma, Epidural, Cranial/ | 3452 |
| 2 | ((epidural or extradural) adj (h?ematoma? or h?emorrhage?)).ti,ab,kf. | 5050 |
| 3 | 1 or 2 | 6319 |
| 4 | surgery.fs. | 2024715 |
| 5 | exp Craniotomy/ | 15984 |
| 6 | (surg* or neurosurg*).ti,ab,kf,jw. | 2811396 |
| 7 | (Craniectom* or Craniotom* or Trephining? or Trepanning? or Evacuation? or Trephination? or Trepanation?).ti,ab,kf. | 37511 |
| 8 | or/4-7 | 3632158 |
| 9 | 3 and 8 | 4138 |
| 10 | trauma severity indices/ or glasgow coma scale/ or glasgow outcome scale/ | 19671 |
| 11 | Treatment Outcome/ or Prognosis/ | 1473978 |
| 12 | mortality/ or fatal outcome/ or hospital mortality/ or survival rate/ | 323826 |
| 13 | (outcome? or mortality).ti,kf. | 555275 |
| 14 | (outcome? or mortality).ab. /freq=5 | 173861 |
| 15 | (glasgow adj2 scale?).ti,ab,kf. | 13188 |
| 16 | or/10-15 | 2065629 |
| 17 | 9 and 16 | 1169 |
| 18 | limit 17 to "humans only (removes records about animals)" | 1160 |
| 19 | exp age groups/ not exp adult/ | 1913365 |
| 20 | 18 not 19 | 993 |
| 21 | (english or german).lg. | 28403734 |
| 22 | 20 and 21 | 888 |
| 23 | Systematic Review.pt. | 145402 |
| 24 | review.pt. | 2765135 |
| 25 | (medline or medlars or embase or pubmed or cochrane or (scisearch or psychinfo or psycinfo) or (psychlit or psyclit) or cinahl or ((hand adj2 search$) or (manual$ adj2 search$)) or (electronic database$ or bibliographic database$ or computeri?ed database$ or online database$) or (pooling or pooled or mantel haenszel) or (peto or dersimonian or der simonian or fixed effect)).tw,sh. or (retraction of publication or retracted publication).pt. | 384942 |
| 26 | 24 and 25 | 167789 |
| 27 | meta-analysis.pt. or meta-analysis.sh. or (meta-analys$ or meta analys$ or metaanalys$).tw,sh. or (systematic$ adj5 review$).tw,sh. or (systematic$ adj5 overview$).tw,sh. or (quantitativ$ adj5 review$).tw,sh. or (quantitativ$ adj5 overview$).tw,sh. or (quantitativ$ adj5 synthesis$).tw,sh. or (methodologic$ adj5 review$).tw,sh. or (methodologic$ adj5 overview$).tw,sh. or (integrative research review$ or research integration).tw. | 357039 |
| 28 | 23 or 26 or 27 | 423114 |
| 29 | 22 and 28 | 25 |
| 30 | randomized controlled trial.pt. or (random$ or placebo$ or single blind$ or double blind$ or triple blind$).ti,ab. or (retraction of publication or retracted publication).pt. | 1419143 |
| 31 | (animals not humans).sh. or ((comment or editorial or meta-analysis or practice-guideline or review or letter) not randomized controlled trial).pt. or ((random sampl$ or random digit$ or random effect$ or random survey or random regression).ti,ab. not randomized controlled trial.pt.) | 9345199 |
| 32 | 30 not 31 | 1038533 |
| 33 | 22 and 32 | 23 |
| 34 | exp cohort studies/ or exp epidemiologic studies/ or exp clinical trial/ or exp evaluation studies as topic/ or exp statistics as topic/ | 5738525 |
| 35 | ((control and (study or group*)) or (time and factors) or cohort or program or comparative stud* or evaluation studies or survey* or follow-up* or ci).mp. | 7588571 |
| 36 | 34 or 35 | 9962120 |
| 37 | (animals/ not humans/) or comment/ or editorial/ or exp review/ or meta analysis/ or consensus/ or exp guideline/ or hi.fs. or case report.mp. | 9234442 |
| 38 | 36 not 37 | 7717504 |
| 39 | 22 and 38 | 513 |
| 40 | case reports/ or (case? not control).ti,kf. | 2621651 |
| 41 | 22 not 40 | 591 |
| 42 | 29 or 33 or 39 or 41 | 692 |

Cochrane Library 15.02.2021

| ID | Search | Hits |
| --- | --- | --- |
| #1 | [mh "Hematoma, Epidural, Cranial"] | 13 |
| #2 | ((epidural or extradural) NEXT (h?ematoma? or h?emorrhage?)):ti,ab,kw | 168 |
| #3 | #1 or #2 | 178 |
| #4 | [mh ^"trauma severity indices"] or [mh "glasgow coma scale"] or [mh "glasgow outcome scale"] | 785 |
| #5 | [mh ^"Treatment Outcome"] or [mh ^"Prognosis"] | 143646 |
| #6 | [mh ^"mortality"] or [mh ^"fatal outcome"] or [mh ^"hospital mortality"] or [mh ^"survival rate"] | 11529 |
| #7 | (outcome? or mortality):ti,ab,kw | 632210 |
| #8 | (glasgow NEAR/2 scale?):ti,ab,kw | 2458 |
| #9 | {or #4-#8} | 638034 |
| #10 | #3 and #9 | 118 |

Epistemonikos 15.02.2021

| Search | Results |
| --- | --- |
| ("epidural hematoma" OR "epidural haematoma" OR "epidural hematomas" OR "epidural haematomas" OR "epidural hemorrhage" OR "epidural haemorrhage" OR "epidural hemorrhages" OR "epidural haemorrhages" OR "extradural hematoma" OR "extradural haematoma" OR "extradural hematomas" OR "extradural haematomas" OR "extradural hemorrhage" OR "extradural haemorrhage" OR "extradural hemorrhages" OR "extradural haemorrhages") AND (outcome* OR mortality OR glasgow) | 75 |
| Filter: Systematic Review | 40 |

Pubmed Similar Articles (based on the first 100 linked references for each article) 15.02.2021

| Search number | Query | Results |
| --- | --- | --- |
| 1 | 8437654 | 1 |
| 2 | Similar articles for PMID: 8437654 | 145 |
| 3 | 9820707 | 1 |
| 4 | Similar articles for PMID: 9820707 | 109 |
| 5 | 19812961 | 1 |
| 6 | Similar articles for PMID: 19812961 | 148 |
| 7 | 29959068 | 1 |
| 8 | Similar articles for PMID: 29959068 | 178 |
| 9 | 8437654 9820707 19812961 29959068 19077617 19262419 23238942 28186445 27598608 6504278 19930556 30796557 9348150 3335912 19812962 17097656 19262419 1411608 21725793 27622712 23384984 10069586 19812955 19262419 23689396 21704911 15518850 21336188 31605850 2915245 19812960 28234186 16273415 29959068 14530763 9348150 17097656 27593740 19262419 22936224 11995217 25073982 14634882 16273415 28409733 8875479 23481903 31288223 23759341 7782824 18493704 25204718 19440005 26722857 1411608 20925589 15686177 31669243 12181688 19077617 26742836 6470802 17465359 20422510 9419033 15296117 23564148 26786639 27663125 10809484 19338415 27663125 8561036 3173664 29567873 26722857 29959068 15795837 18695475 28409733 8402704 1237759 24773559 23013227 30478538 24773559 19812956 27890762 17639868 11070432 18393161 19440005 8858030 14589226 9816666 24458036 24440624 10029038 19077617 23161150 17061134 1886660 14663560 25073982 8128257 16007322 22543444 24773559 21336188 9657190 23546923 28867312 8532132 8442541 22727212 22678529 30796557 19812961 9688114 11995217 20108539 21160234 21615250 28894943 1411608 557748 19079389 21704911 2915245 17097656 15739038 30797192 21243247 18727335 25073982 25096980 24905840 17766433 22327713 27593740 25237431 21039137 23384251 15626831 6728158 23238942 16904897 24905840 27385774 24410128 26351872 29148640 15795837 1694269 20517764 17639868 31669243 25393339 23564139 25301742 1603217 7754821 23564117 25050450 22274969 19077617 22769066 26742836 25557102 2588898 25393339 28049032 32553454 9113968 27476687 9974064 8475807 2295916 22762248 8437654 10029038 9457718 27235128 31669243 3723178 7666220 20669113 26875660 25050450 8437654 26872068 26495949 18325427 28894943 22216933 23769388 20223405 8858030 23290122 17061134 28894943 24905840 20452786 3228007 23013227 27598608 22377637 29614364 2295916 10421184 23865130 21039137 14643923 21961552 29172712 30074459 2342147 26140392 19061378 29304855 28234186 8499326 23111437 27392896 23588913 9267956 24506250 10029038 25393339 18022491 31096029 28945629 7753416 9760981 23634922 24732678 11794591 2399732 19065118 28055227 26722857 23384984 27385774 23759341 15287484 27663125 12865006 9820707 14762615 11373416 23397272 26140392 20422510 15220504 30071335 11794591 10940767 23769388 1605089 19463048 31177323 20925589 24124711 20713606 8319240 25058258 20182201 23746127 9974064 27890762 24831363 31177323 27593740 31288223 20804513 32762545 31071445 7241184 26875660 23689396 27255655 22182869 28894943 26351872 10809484 3880412 26090828 30385362 3228007 5425151 23013227 28776222 8697457 23079406 28055227 26855305 2263991 7846277 24848173 29772371 20713606 14963742 10809484 25955872 22120266 3614604 27149021 23200264 3335912 14505243 28688799 22909250 25096980 20422510 22274969 22422166 24773559 23161150 29545220 28194757 9454977 8147252 23564146 23564119 18288440 1590183 30892670 29419604 9820707 16159521 21165542 17318751 18727335 19698050 22402877 25361494 20925589 7424317 15494119 25393339 19212694 1413279 26854773 27215665 9792964 9348150 24275779 15795837 16710967 27385774 22665001 18841222 24948502 28962067 1123660 26287640 12391460 26351872 11780896 25803654 7666220 6494859 22327712 27739945 32925745 8676403 27593740 8858030 28427975 22120266 23809681 27032920 2399732 7753416 11482699 24662876 28922710 9005573 9820707 28430697 16138273 7331863 9348150 32955907 31288223 22735059 28962067 32925745 25073982 7411669 23239253 15626829 15227846 7754822 6504278 23384984 29673818 9202765 6514123 20699736 15626831 22737301 22678529 27385774 | 294 |
| 10 | #9 NOT ("Animals"[Mesh] NOT "Humans"[Mesh]) | 293 |
| 11 | #10 NOT ("Age Groups"[Mesh] NOT "Adult"[Mesh]) | 226 |
| 12 | #11 AND ("english"[Language] OR "german"[Language]) | 212 |
| 13 | #12 AND systematic[sb] | 1 |
| 14 | #12 AND (randomized controlled trial[Publication Type] OR (random*[Title/Abstract] AND controlled[Title/Abstract] AND trial[Title/Abstract])) | 4 |
| 15 | #12 AND (cohort[all] OR (control[all] AND study[all]) OR (control[tw] AND group*[tw]) OR epidemiologic studies[mh] OR program[tw] OR clinical trial[pt] OR comparative stud*[all] OR evaluation studies[all] OR statistics as topic[mh] OR survey*[tw] OR follow‐up*[all] OR time factors[all] OR ci[tw]) NOT ((animals[mh:noexp] NOT humans[mh:noexp]) OR comment[pt] OR editorial[pt] OR review[pt] OR meta analysis[pt] OR case report[tw] OR consensus[mh] OR guideline[pt] OR history[sh]) | 165 |
| 16 | #12 NOT ("Case Reports" [Publication Type] OR (case*[ti] NOT control[ti])) | 171 |
| 17 | #13 OR #14 OR #15 OR #16 | 187 |

ClinicalTrials.gov 15.02.2021

| Search | Studies |
| --- | --- |
| Other terms: glasgow \| Condition: (epidural OR extradural) AND (hematoma OR haematoma OR hemorrhage OR haemorrhage) | 4 |
